# A Conceptual Framework for Mapping Quantitative Trait Loci Regulating Ontogenetic Allometry

**DOI:** 10.1371/journal.pone.0001245

**Published:** 2007-11-28

**Authors:** Hongying Li, Zhongwen Huang, Junyi Gai, Song Wu, Yanru Zeng, Qin Li, Rongling Wu

**Affiliations:** 1 Department of Statistics, University of Florida, Gainesville, Florida, United States of America; 2 National Center for Soybean Improvement, Nanjing Agricultural University, Nanjing, Jiangsu, People’s Republic of China; 3 Department of Agronomy, Henan Institute of Science and Technology, Xinxiang, Henan, People’s Republic of China; 4 School of Forestry and Biotechnology, Zhejiang Forestry University, Lin’an, Zhejiang, People’s Republic of China; Michigan State University, United States of America

## Abstract

Although ontogenetic changes in body shape and its associated allometry has been studied for over a century, essentially nothing is known about their underlying genetic and developmental mechanisms. One of the reasons for this ignorance is the unavailability of a conceptual framework to formulate the experimental design for data collection and statistical models for data analyses. We developed a framework model for unraveling the genetic machinery for ontogenetic changes of allometry. The model incorporates the mathematical aspects of ontogenetic growth and allometry into a maximum likelihood framework for quantitative trait locus (QTL) mapping. As a quantitative platform, the model allows for the testing of a number of biologically meaningful hypotheses to explore the pleiotropic basis of the QTL that regulate ontogeny and allometry. Simulation studies and real data analysis of a live example in soybean have been performed to investigate the statistical behavior of the model and validate its practical utilization. The statistical model proposed will help to study the genetic architecture of complex phenotypes and, therefore, gain better insights into the mechanistic regulation for developmental patterns and processes in organisms.

## Introduction

An incredible diversity has been observed in the scaling relationships among different body parts or traits, and between these and overall body size [1]–[7]. The differentiation in such allometries among traits has been thought to be a driving force by which morphology evolves [8]. Perhaps the most fundamental allometric relationship is the one that relates physiological, morphological and anatomical attributes with body size [1], [2], [9], [10]. Interestingly, the preponderance of data suggests that many metabolism-related structural traits scale as multiples of one quarter of body size [11], rather than one third as expected from Euclidean geometric scaling. Despite some vigorous debate [12]–[14], the quarter-power allometric scaling has been regarded as a universal phenomenon in biology, explained from fundamental principles of biology and biophysics [15]–[18]. However, even with over a century of interest in the evolution of allometry, essentially nothing is known about the genetic and developmental mechanisms of differentiation in allometric scaling relationships, although developmental processes must have played a central role in maintaining the functional scaling relationships among traits as well as in their evolution [6], [19], [20].

The past two decades have witnessed a surge of interest in applying geometric morphometric approaches to understand how body shape changes and how such a change is associated with allometry during ontogeny [21]–[23]. For instance, these approaches have been used to study the ontogeny of body shape change for a few number of fishes [24], [25], showing that body shape changes during ontogeny are not simply the result of uniform large-scale events but that localized small-scale shape changes contribute to its ontogeny. However, none of these studies have attempted to detect the genetic machinery for ontogenetic allometry from a developmental perspective.

One promising approach is to characterize specific genetic variants that regulate the ontogeny of allometry and compare them with those genetic variants that determine body size [26]. The feasibility of this approach results from two recent significant developments. First, the progress of whole genome sequence projects in microbes, plants, animals and human beings provides fundamental information about the organization and structure of genomes and proteins [27]. Second, the availability of powerful statistical methods allows direct association studies between genetic variants and complex metabolic processes [28]–[37]. Among these methods, a full-dimensional analysis of multiple traits can map and estimate quantitative trait loci (QTLs) for trait correlations [38]–[41]. However, these multi-trait mapping approaches cannot take advantages of ontogenetic allometry, and will thus be less powerful than an approach that specifically incorporates allometry. Further, these methods do not extend easily to many time points or to missing data because of computational burden and estimation instability [42]. By incorporating the mathematical aspects of allometric scaling into the mixture model-based framework, Wu and group developed a series of conceptual models and computational algorithms for detecting QTLs that govern allometry and testing the hypotheses about the genetic control of allometry [43]–[46]. However, there is a serious lack of sophisticated models that have power to detect genetic variants responsible for the combined effects of size and development on allometric attributes at different organizational levels.

In this article, we will frame a general genetic model for explaining universal allometric scaling laws and derive a statistical algorithm for detecting particular QTLs that contribute to these laws. The model embeds the allometric power equation into the framework for functional mapping constructed to map a dynamic trait [26], allowing the identification of QTLs that determine the degree and pattern of the response of a body part to body size in development. The new model can be readily extended to predict how the structure and functioning of a biological system are affected by genetic interactions derived from different regions of the genome. The utilization of the model has been tested and validated by analyzing real data from the soybean genome project, in which several significant QTLs were detected on different soybean chromosomes to affect the allometric scaling between stem and whole-plant biomass during development. The empirical power of the model and the precision of its parameter estimation for a practical data set have been investigated through computer simulation studies. The model will provide a quantitative framework for analyzing the genetic architecture of ontogenetic changes in shape and allometry.

## Methods

### Allometry

Consider a simple backcross or recombinant inbred line (RIL) design in which *n* progeny are segregating in a 1∶1 ratio at each locus. A genetic linkage map, aimed to identify segregating quantitative trait loci (QTL), is constructed with polymorphic markers genotyped through the genome. All the progeny are measured for two developmentally related traits, Z and Y, at *T* time points, (*t*_1_,…*t_T_*). If two traits of a similar developmental origin can be modeled by an allometric equation, this can be expressed as
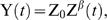
(1)where *t* denotes a particular time, Z_0_ is a normalization constant and β is a scaling exponent. Taking log-transformation at both sides of Equation 1, the allometric equation is linearized as
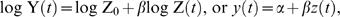
(2)where
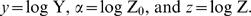
We assume that the log-transformed traits at time *t* have a normal distribution. The observations for the two traits at time *t* can be expressed as
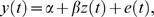
where *e*(*t*) is the measurement error at time *t* following *N*(0, σ^2^(*t*)).

**Figure 1:**
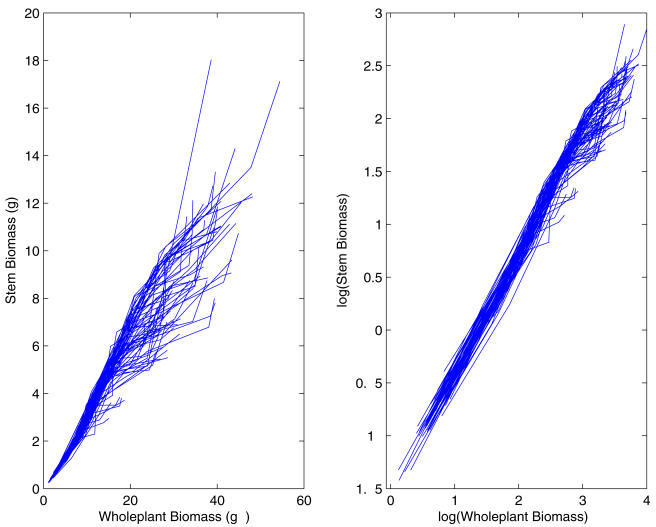
The allometric scaling relationship between stem biomass and whole-plant biomass for the RILs from a soybean mapping population.

Figure 1 plots two allometrically related traits, stem biomass and whole-plant biomass, during development for soybean plants randomly sampled from an RIL population, in which the original power relationship (Fig. 1A) is straightened out after log-log transformation (Fig. 1B). Such a log-linear allometric relationship has been justified from fundamental biological principles [15]–[18].

### Likelihood

For each of the two dynamic traits, *z*(*t*) and *y*(*t*), functional mapping has established a general statistical framework for mapping its underlying QTLs with molecular markers [47]. In this study, we will incorporate the allometric scaling law (Equation 1) into functional mapping. But different from the previous treatment for bivariate functional mapping by Wu and Hou [45], we will found functional mapping on the dependent trait, connected with the independent trait by the allometric equation. To simplify the description of our model, we assume that one single QTL is involved in the allometric control. The derivation of a more realistic multiple-QTL model is conceptually straightforward with the idea of the one QTL model, but this extension raises many statistical issues, such as model selection for the optimal number of QTL involved (see ref. [33]).

Similar to Ma et al. [47], we formulate the likelihood function for one dynamic trait, y, controlled by a QTL bracketed by a pair of marker (M), as
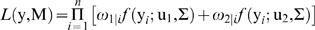
(3)where y*_i_* = (*y_i_*(*t*_1_),…,*y_i_*(*t_T_*))′ is the observation vector, (*t*_1_,…,*t_T_*)′ denote the time points when the observations are measured, *f*(y*_i_*;u*_j_*, Σ) is the multivariate density function for different QTL genotypes (subscripted by *j* = 1 for *QQ* or 2 for *qq*) with mean vector u*_j_* = (*u_j_*(*t*_1_),…*u_j_*(*t_T_*))′ and time-dependent covariance matrix Σ, and ω_1|*i*_ and ω_2|*i*_ are the conditional probabilities of a QTL genotype, 1 or 2, given the genotype of progeny *i* for two flanking markers. The conditional probabilities are expressed in terms of the recombination fractions (for the backcross design) or the proportions of recombinant homozygotes (for the RIL design) [48] between the left marker and QTL (*r*_1_) and between the QTL and right marker (*r*_2_).

### Modelling the Mean Vector

If the allometric relationship between two biological traits is controlled by a QTL, the linearized power equation (2) can be used to model the genotypic mean vector in the likelihood (3) with genotype-specific parameter sets (α_1_, β_1_) or (α_2_, β_2_). Thus, by testing the difference between these two parameter sets, we can conclude whether there is a specific QTL for allometric scaling and how the QTL controls the scaling relationship. Wu and Hou [45] modeled the allometric scaling relationship by incorporating the genotypic vectors of the two traits, *y* and *z*, i.e.,
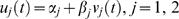
(4)where *u_j_*(*t*) and *v_j_*(*t*) are the genotypic values of traits *y* and *z* for QTL genotype *j* at time point *t*. This treatment needs to simultaneously estimate the genotypic vector of traits *y* and *z*, expressed as (*u_j_*(*t*_1_),…, *u_j_*(*t_T_*), *v_j_*(*t*_1_),…, *v_j_*(*t_T_*)). More importantly, the time-dependent covariance matrix for each trait and the time-dependent covariance matrix between the two traits need to be specified at a time, leading to a double-sized covariance matrix of dimension 2T×2T. All these will largely increase the number of parameters to be estimated, making the computation quickly prohibitive and the parameter estimation imprecise.

To overcome this problem, we will formulate a different model for the allometric relationship. Given that the allometric change of one trait is not only regulated by the underlying genes, but also by physiology-and metabolism-related characteristics that contain the influences of both genes and environments [7], we model the genotypic vector of a trait with the phenotypic value of a second allometrically related trait. Thus, Equation 4 is changed as
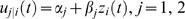
(5)where *u_j_*_|*i*_(*t*) = E[*y_i_*(*t*)|*Z_i_*(*t*), QTL genotype *j*]. This can be explained from genetic and statistical perspectives. In genetics, Equation 5 states how the genotypic value of trait *y* for QTL genotype *j* scales as, or responds to, the phenotypic change of trait *z* during ontogeny. If parameter set (α*_j_*, β*_j_*) is not different between QTL genotypes, this means that this QTL does not determine the allometric scaling between traits *y* and *z*. Equation 5 indicates that given the QTL genotype we can regress *y* on the covariate *z* using a linear regression model.

### Modeling the Time-Dependent Covariance

The residual covariance matrix, Σ, generally follows an autocorrelation structure, which can be mathematically modeled. A number of statistical models, such as autoregressive [49] and antedependent [50] models, have been formulated to model such a structure. In Zimmerman and Núñez-Antón [51], the advantages of structured antedependent (SAD) model have been extensively discussed, which include (1) the assumptions of variance and correlation stationary are not needed, and (2) closed forms exist for the inverse and determinant of the SAD matrix.

For first order SAD models with an antedependence parameter ρ, if we assume the innovation variances σ^2^(*t*) to be a constant σ^2^ over time, explicit forms of variance and correlation functions can be obtained as
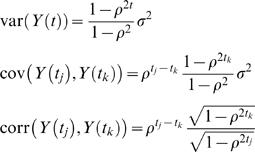
for *t_k_*≥*t_j_*. For this simplest SAD(1) model, the variance and correlation functions are non-stationary. They change as time and time interval change.

### Computational Algorithm

When Equation 5 is substituted into the likelihood of Equation 3, the likelihood function is now expressed as
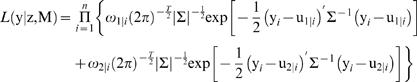
(6)where u_*j*|*i*_ = (*u_j_*_|*i*_(*t*_1_),…, *u_j_*_|*i*_(*t_T_*))′(*j* = 1, 2) are the QTL genotype-specific mean vectors which also depend on the trait *z* (see Equation 5).

The underlying unknown parameters are composed of (*r*_1_ or *r*_2_, α*_j_*, β*_j_*, ρ, σ^2^). In the Appendix S1, we derive the EM algorithm to obtain the maximum likelihood estimates (MLEs) of these unknown parameters. The estimates of the sampling errors of the MLEs can be obtained from Louis’ [52] approach derived within the context of a mixture model.

The heritability of trait *y* at time *t* is calculated as follows:
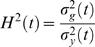
where
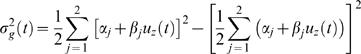
and
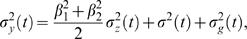
with
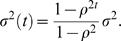
Here *u*_z_(*t*) and σ^2^(*t*) are the mean and variance for trait *z* at time *t*, respectively, which can be estimated from observations.

### Hypothesis Testing

As shown in our previous publications [26], [43], [47], [53], functional mapping is advantageous for the tests of biologically meaningful hypotheses regarding genetic actions and organ development. Here, we outline several important hypotheses for the genetic control of allometric scaling. The first hypothesis is about the existence of QTL, which can be tested by formulating the null hypothesis,

H_0_: α_1_ = α_2_ and β_1_ = β_2_

H_1_: At least one of the equalities in H_0_ does not hold.

The log-likelihood ratio statistic is calculated as
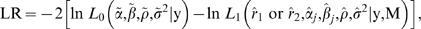
(7)where the tildes and hats are the MLEs of parameters under the null and alternative hypotheses, respectively. The LR value is then compared with the critical threshold determined from permutation tests, as advocated by Churchill and Doerge [54], to test the significance of the QTL hypothesized.

We are also interested in the genetic cause for the differentiation in ontogenetic allometric scaling. This can be investigated by testing the normalization (α) and exponent constant (β) individually. Some study suggests that α is a characteristic of species or populations [10], whereas a recent survey by Niklas and Enquist [55] shows that all plants have a similar normalization constant and, therefore, comply with a single allometric formula. This debate can be solved by testing whether α equals to a specific constant for different plant species.

In practice, the exponent coefficient β can be considered as a constant if the allometric relationship of the two traits studied is known. For example, body length scales as the 1/4-power of body mass [15], [16]. In this case, β = 1/4 can be directly substituted into Equation 5 to obtain estimates for the remainder of the unknown parameters. Owing to the reduced number of the unknowns to be estimated, such a substitution can potentially increase the precision and power of parameter estimation.

All the tests for α and β can be performed by calculating a likelihood ratio statistic which asymptotically follows a chi-square distribution with the corresponding degrees of freedom. In actual data analyses, an empirical approach based on simulation studies can be used to determine the threshold for these tests.

## Results

### A Worked Example

We used a real example from the soybean genome project to validate the model proposed for mapping ontogenetic allometry. Two original inbred lines of soybean, Kefeng No. 1 and Nannong 1138-2, as parents were crossed to generate an F_1_ population which was selfed for 7 generations to produce an RIL population composed of two groups of homozygous genotypes each containing two identical alleles from a different parental line. Let 1 and 2 denote the homozygotes derived from the Kefeng No. 1 alleles and Nannong 1138-2 alleles, respectively. A total of 184 RILs were genotyped for 488 molecular markers (restricted fragment length polymorphisms, simple sequence repeats and amplified fragment length polymorphisms) that construct a linkage map with 25 linkage groups covering 4,151.2 cM of the soybean genome [56].

The RILs were planted in a simple lattice design with multiple replicates in a plot at Jiangpu Station, Nanjing Agricultural University, Nanjing, China. The plants were harvested to measure their above- and under-ground biomass for eight times with the first time at the 28th day after emergence and successive seven times every 10 days thereafter. For the same RIL, the phenotypic values measured for different times correspond to successive measurements on a time scale. In this study, we will analyze the genetic control of the ontogenetic allometric scaling relationship between stem and whole-plant biomass.

As shown by a subset of RILs from the mapping population in Figure 1, stem biomass scales as a power function of whole-plant biomass. The Pearson correlation coefficients between the two log-transformed traits from the samples for all subjects are all close to 1, ranging from 0.9381 to 0.9992. The sample mean of the coefficients for all individuals is 0.9885 with a sample standard error 0.0092. Thus, a high linear trend between the log-transformed stem biomass and whole plant biomass is appropriate for our allometric mapping model.

**Figure 2:**
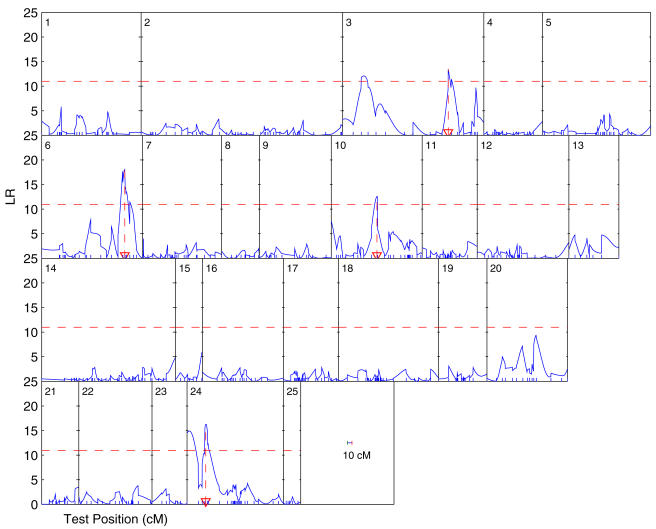
The LR profile of the likelihoods under the null (there is no QTL) and alternative hypothesis (there is a QTL) across the lengths of 25 chromosomes for the allometric scaling relationship between stem and whole-plant biomass growth trajectories in a soybean RIL population. The 5% significance critical threshold (10.98) determined from 1000 permutation tests is indicated by the broken horizontal line. The arrowed broken vertical line indicates the MLE of the QTL location.

This relationship was incorporated into functional mapping to characterize specific QTL that control the allometric change of stem biomass relative to whole-plant biomass. Different from the backcross population, the conditional probabilities of a QTL genotype given marker genotypes are expressed in terms of the proportion of recombinant homozygotes [57]. We detected five significant allometry QTLs, two located between markers GMKF082c and GMKF168b and at marker A520T on chromosome 3, one located between markers GMKF059a and satt319 on chromosome 6, one located between markers Satt372 and Satt154 on chromosome 10, and one between markers GMKF082b and satt331 on chromosome 24, as indicated by peaks of the LR profile beyond the 5% critical threshold obtained from 1000 permutation tests (Fig. 2). We tend to claim two QTL on chromosome 3 in this particular example (although they were not tested simultaneously) because their detected positions are about 120 cM apart, suggesting an unlinked relationship. The permutation tests [54] were performed by repeatedly reshuffling stem biomass and whole-plant biomass among different RIL progeny but leaving marker genotypes unchanged. It is interesting to point out that the QTL on chromosome 6 is located between two closely spaced markers (5 cM apart), with 4 cM to the left marker and 1 cM to the right one. In conjunction with a narrow LR peak, this suggests that the detection of this QTL has a high resolution. Two QTLs were counted on chromosome 3 because they are distant enough from each other to infer the existence of two unlinked QTLs. On chromosome 24, there are two well-separated peaks, but they are two close to claim the existence of two different QTL. Thus, we only counted one QTL with a higher peak. In the Discussion, we will provide a possible solution into the test of two linked QTLs under the framework of allometry QTL mapping by implementing the idea of composite interval mapping [29], [30].

**Figure 3:**
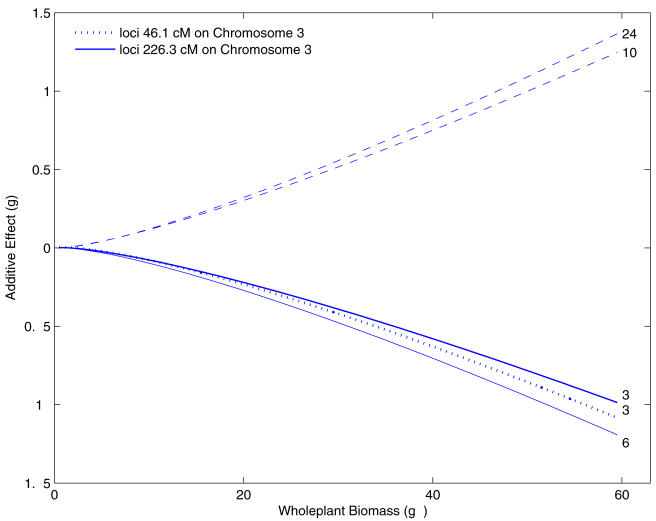
Body size-dependent additive genetic effects calculated from ontogenetic allometry curves for two different genotypes at each of the five QTL detected on chromosomes 3, 6,10 and 24.

The model provided the MLEs of genotype-specific curve parameters and covariance-structuring SAD parameters when each of the significant QTL was detected (Table 1). These estimates display great precision, as reflected by their small sampling errors estimated by Louis’ approach [52]. The estimated genotypic power curve parameters are used to calculate additive genetic effects, *a*(*t*), at each QTL that vary with time-dependent whole-plant biomass by
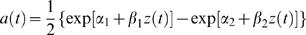
for an RIL design. The positive value of *a*(*t*) implies that parent Kefeng No. 1 contributes favorable alleles to increased stem biomass, whereas the negative value corresponds to the favorable contribution made by parent Nannong 1138-2. As shown by Fig. 3, the additive effects of each QTL on stem biomass change with whole-plant biomass. Based on their signs, it is suggested that at the two QTLs on chromosomes 10 and 24 favorable alleles for increased stem biomass are contributed by parent Kefeng No. 1, whereas the inverse pattern is true for the three QTLs on chromosomes 3 and 6. We estimated the heritability of each QTL for stem biomass at the fifth time point, which ranges from 0.0198 to 0.0381 for the five QTL detected at different chromosomes.

**Table 1:** Maximum likelihood estimates (MLEs) of genotype-specific power parameters (*α* and *β*) for each QTL detected and SAD(1) parameters (ρ and σ^2 ^) that model the covariance structure.

	Chromosome (Position)									
	3 (46.1)		3 (226.3)		6 (178.3)		10 (98)		24 (40.2)	
	*α*	*β*	*α*	*β*	*α*	*β*	*α*	*β*	*α*	*β*
*QQ*	−1.4653 (0.0088)	1.0268 (0.0046)	−1.4728 (0.0089)	1.0276 (0.0053)	−1.4735 (0.0090)	1.0251 (0.0046)	−1.4714 (0.0096)	1.0673 (0.0053)	−1.4750 (0.0075)	1.0605 (0.0045)
*qq*	−1.4958 (0.0087)	1.0666 (0.0045)	−1.4894 (0.0073)	1.0614 (0.0040)	−1.4859 (0.0075)	1.0640 (0.0026)	−1.4875 (0.0126)	1.0347 (0.0088)	−1.4819 (0.0081)	1.0304 (0.0052)
*ρ*	0.7113 (0.0248)		0.7189 (0.0235)		0.7159 (0.0253)		0.6820 (0.0333)		0.7036 (0.0212)	
*σ* ^2^	0.0192 (0.0003)		0.0190 (0.0003)		0.0182 (0.0003)		0.0183 (0.0004)		0.0188 (0.0002)	
LR	12.0794		13.4529		18.0394		12.5744		16.2926	
Genome-wide threshold (5%)					10.9817					

The numbers in the parentheses are estimated standard errors for the MLEs.

The position of a detected QTL is expressed as the genetic distance (in cM) from the first marker of a chromosome (see Fig. 2).

### Computer Simulation

Monte Carlo simulation studies were performed to examine the reliability of the parameters estimates in the soybean example above by mimicking the data structure of the mapping population. Also, additional simulation analyses were used to investigate the statistical properties of the model in terms of estimation precision and power under different sample sizes and heritability levels. An RIL population was simulated for 11 equally spaced markers that construct a linkage group of length 200 cM. A QTL that affects the ontogenetic allometric scaling relationship between traits *y* and *z* is assumed at 85 cM from the first marker. The data for marker genotypes were simulated in terms of the recombinant homozygote proportion (R). The genetic distances between markers are calculated from the recombination fractions (r) with the Haldane map function. The recombination fractions were calculated from the recombinant homozygote proportions using
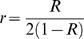
Dynamic trait *y* is assumed for each RIL plant to follow a multivariate normal distribution with mean vector specified by Equation 5 and covariance matrix specified by the SAD model. Dynamic trait *z* is also assumed to follow a multivariate normal distribution with time-increasing means and SAD-structured covariance matrix. And observations of traits *y* and *z* are obtained at 8 time points (1,…, 8). The innovative variance for trait *y* is determined by assuming different heritability levels (*H* ^2^ = 0.1 and 0.4) at the nearly middle period (time point 5) of time course. The size of heritability reflects the contributions of other unobserved genes to phenotypic variation as well as the influences of measurement errors that cause observations to deviate from allometry. Thus, a small heritability is partially associated with a large degree of deviation from allometry. Two different sample sizes (n = 100 and 400) are considered for the RIL population.

**Figure 4:**
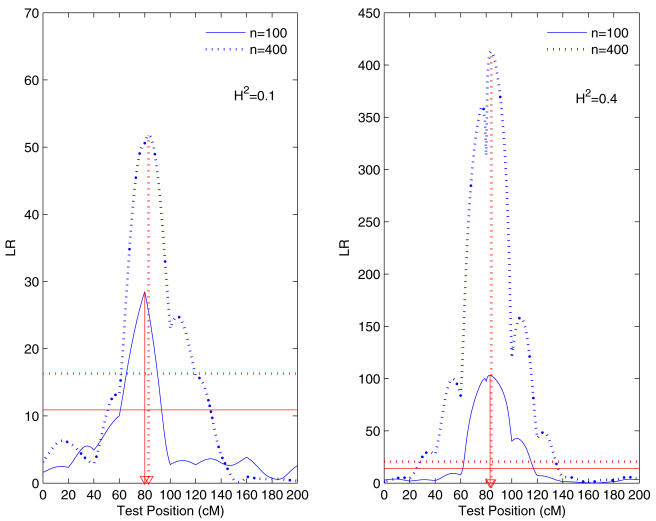
The LR plots for the simulated data under different sample sizes (n = 100 and 400) and heritabilities (*H* ^2^ = 0.1 and 0.4).

The ontogenetic allometric functional mapping was used to map QTL for the simulated data. Figure 4 illustrates the LR profiles for the data simulated under different heritability levels and sample sizes. In general, the location of the QTL can be well estimated even for a small sample size (100) and heritability (0.1). The estimation accuracy of the QTL location can be increased when the sample size increase to 400 and or the heritability increases to 0.4. The MLEs of the curve parameters and covariance-structuring parameters are tabulated in Table 2. All the parameters can be reasonably estimated as indicated by small standard errors, with increased estimation precision associated with increased sample sizes and heritabilities. When the sample size and heritability are small (100 and 0.1), the power to detect a significant allometry QTL is reasonably high, and can increase dramatically when *n* increases to 400 and/or when *H* ^2^ increases to 0.4.

**Table 2:** Averaged MLEs of the parameters (with standard errors given in the parentheses) based on 400 simulation replicates under different simulation schemes combining different heritabilities (*H* ^2^) and sample size (*n*).

Scheme			*QQ*		*qq*				
*H* ^2^	*n*	Position	*α*_1_	*β*_1_	*α*_2_	*β*_2_	*ρ*	*σ* ^2^	Power
True value	85	−1.2	0.7	−1.4	0.5	0.7	0.1472		
0.1	100	84.84 (16.20)	−1.198 (0.052)	0.702 (0.047)	−1.398 (0.053)	0.499 (0.049)	0.695 (0.031)	0.146 (0.008)	0.88
0.1	400	84.61 (3.14)	−1.201 (0.025)	0.699 (0.02)	−1.401 (0.024)	0.50 (0.022)	0.698 (0.015)	0.146 (0.004)	1
True value	85	−1.2	0.7	−1.4	0.5	0.7	0.0197		
0.4	100	84.99 (2.49)	−1.200 (0.016)	0.699 (0.014)	−1.400 (0.016)	0.500 (0.015)	0.695 (0.032)	0.020 (0.001)	1
0.4	400	85.06 (1.10)	−1.199 (0.008)	0.700 (0.008)	−1.400 (0.009)	0.499 (0.008)	0.698 (0.017)	0.020 (0.001)	1

The power was empirically calculated as the percentage of the number of simulation replicates, in which significant QTL is detected, over the total number of simulation replicates.

## Discussion

The term allometry that describes scaling relationships between different organ parts can be understood from three different perspectives: static, ontogenetic and evolutionary [19], [58]. Static allometry refers to the scaling among individuals between two different traits after growth has ceased or at a particular developmental stage. Ontogenetic allometry is the growth trajectory of one trait relative to the other (i.e., shape) during an individual’s lifetime. Evolutionary (or phylogenetic) allometry is the size relationship between traits across species. Much earlier work has focused on the developmental processes and constraints that shape static allometry [5] as well as on the evolution of allometries [8]. With the recognition of development as an evolutionary factor, evolutionary developmental biology (evo-devo) has revived an interest in understanding the process of evolution [59]. It is anticipated that ontogenetic allometry that determine the direction and pattern of development will be a component of primary importance to construct the evo-devo framework.

Although allometry has been an important subject of biological research for over a century, little is known about the mechanism of its genetic control. Recent genomic technologies have opened a new avenue to generate genome-wide marker data and, therefore, characterize the specific loci or DNA sequence variants that are associated with the phenotypic variation in static [28]–[30], [37] or dynamic traits [26]. In this article, we have developed a statistical model for deploying such technologies to map quantitative trait loci (QTLs) that are responsible for ontogenetic allometry. This model allows for the characterization of genetic loci that cause ontogenetic shape change and transformations during growth and development.

The model for mapping ontogenetic allometry is built on the foundation of functional mapping [47], aimed to map QTL that control growth trajectories of a trait. Yet, the new model is different from conventional functional mapping, in which a different but allometrically related trait is embedded through the power equation within the mean vector as a covariate in terms of statistical definition. The function of such embedment is to directly characterize specific QTL that determine ontogenetic changes of allometry and push the hypothesis tests at the interface between genetic actions and shape development. The approach for treating ontogenetic allometric scaling in this article is different from that published in Wu and Hou [45] who jointly modeled two different growth trajectories. Because the current approach only needs to model the relationship of the growth trajectory of two traits, it is more efficient and precise in parameter estimation and computation than Wu and Hou’s approach.

The proposed model has been tested through simulation studies. It is possible that this model can provide the reasonable estimation of the underlying parameters when a trait trajectory has a modest heritability, i.e., with non-genetic variation outweighing genetic variation. In practice, when a trait has a relatively low heritability (e.g., 0.10), a sample size of 400 is recommended to provide satisfactory precision for parameter estimation and power for QTL detection. Our simulation did not test the influence of deviation from allometry on parameter estimation and power. In a similar dynamic genetic study, Yap et al. [60] found that such an influence can be significant but can well be compensated by using a large sample size. This model was used to analyze a real example from the soybean genome project [56] in which there exists a strong linear trend between stem and whole-plant biomass, leading to the detection of five significant QTLs that control ontogenetic allometric scaling between these two traits.

The biological relevance of our model can be enhanced by incorporating the growth equation into the mean vector. Empirical studies on the basis of the goodness-of-fit of observational data suggest that growth can be described by a logistic curve [61], which has been justified by fundamental biological principles [62]. If a logistic equation is used to describe the growth trajectory of trait *z*, we can estimate the curve parameters for each individual, using non-linear least-squares approach, on the basis of
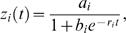
where parameter set (*a_i_*, *b_i_*, *r_i_*) define the curve shape of an individual *i*. These estimates are then substituted into Equation 5 for genotype-specific scaling relationships, which is expressed as
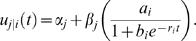
(8)The advantage of Equation 8 lies in its capacity to test the relationship between ontogenetic allometry and growth trajectory through the implementation of growth equation.

The model presented in this article illustrates the idea of mapping the ontogenetic allometry of different biological traits by assuming one underlying QTL. A more sophisticated model that involves multiple QTL and their interactions in a genetic network can be derived with this idea (see [53]) although it needs more extensive computation and model selection [33]. The model described here assumes a full marker data set, but it can be readily modified to consider missing marker data based on a hidden Markov model as advocated by Jiang and Zeng [48]. Also, when phenotypic data are missing at arbitrary time points, the measurement schedule will become unequally spaced. Hou et al. [63] derived an approach for modeling the structure of a longitudinal covariance matrix containing unequal spaced time points, which can be used in this allometric mapping model.

In our example, more than one LR peak was detected on the same chromosome 6. We claimed the existence of two different QTL because they seem to be far enough from each other. However, more precise determination of multiple QTL should be based on multiple interval mapping as proposed by [32]. In particular, Yandell and colleagues constructed a series of Bayesian models that are shown to be powerful for the determination of multiple QTL at the same time [34], [35]. These advanced genetic mapping approaches will stimulate our incorporation to build up a practically more useful allometry mapping framework. Our model can be extended to incorporate the effects of other factors on ontogenetic allometry. For example, in animals, a significant relationship occurs between reproductive status and ontogenetic shape change [21]–[23]. Within both males and females, reproductive classes had significantly different body shapes and in females the trajectories of shape change among reproductive classes were significantly different. By incorporating sexes into the model, sex-dependent QTL for ontogenetic shape changes can be estimated and tested.

Allometric shape changes in development may reflect functional changes and possible relationships between morphology and environment. It is straightforward to incorporate environmental factors into the allometry model to test the genetic effects of QTL on such relationships. The inclusion of multiple environments, as reported in [64], will allow investigating the environment-dependent expression of this allometry QTL.

## Supporting Information

Appendix S1 (0.09 MB DOC)

## References

[1] Kleiber M (1932). Body size and metabolism.. Hilgardia.

[2] Calder WA (1984). Size, Function, and Life History.

[3] West GB, Brown JH, Enquist BJ (1999). A general model for the structure, and allometry of plant vascular systems.. Nature.

[4] Enquist BJ, Niklas KJ (2001). Invariant scaling relations across tree-dominated communities.. Nature.

[5] Enquist BJ, Niklas KJ (2002). Global allocation rules for patterns of biomass partitioning across seed plants.. Science.

[6] West GB, Brown JH (2005). The origin of allometric scaling laws in biology from genomes to ecosystems: towards a quantitative unifying theory of biological structure and organization.. J Exp Biol.

[7] Niklas KJ (2006). A phyletic perspective on the allometry of plant biomass-partitioning patterns and functionally equivalent organ-categories.. New Phytologist.

[8] Frankino WA, Zwaan BJ, Stern DL, Brakefield PM (2005). Natural selection and developmental constraints in the evolution of allometries.. Science.

[9] Peter RH (1983). The Ecological Implications of Body Size.

[10] Niklas KJ (1994). Plant Allometry: The Scaling of Form and Process.

[11] Savage VM, Gillooly JF, Woodruff WH, West GB, Allen AP, Enquist BJ, Brown JH (2004). The predominance of quarter-power scaling in biology.. Fun Eco.

[12] Riisgard HU (1998). No foundation of a “3/4 power scaling law” for respiration in biology.. Ecol Let.

[13] Dodds PS, Rothman DH, Weitz JS (2001). Re-examination of the 3/4-law of metabolism.. J Theor Biol.

[14] White CR, Seymour RS (2003). Mammalian basal metabolic rate is proportional to body mass.. Proc Natl Acad Sci USA.

[15] West GB, Brown JH, Enquist BJ (1997). A general model for the origin of allometric scaling laws in biology.. Science.

[16] West GB, Brown JH, Enquist BJ (1999). The fourth dimension of life: Fractal geometry and allometric scaling of organisms.. Science.

[17] Banavar JR, Maritan A, Rinaldo A (1999). Size and form in efficient transportation networks.. Nature.

[18] Banavar JR, Damuth J, Maritan A, Rinaldo A (2002). Supply-demand balance and metabolic scaling.. Proc Natl Acad Sci USA.

[19] Stern DL, Emlen DJ (1999). The developmental basis for allometry in insects.. Development.

[20] Rombough P (2003). Development rate-Modelling developmental time and temperature.. Nature.

[21] Bookstein FL (1991). Morphometric Tools for Landmark Data: Geometry and Biology.

[22] Rohlf FJ (1998). On applications of geometric morphometrics to studies of ontogeny and phylogeny.. Syst Biol.

[23] Zelditch ML, Swiderski DL, Lundrigan BL (1998). On applications of geometric morphometrics to studies of ontogeny and phylogeny: a reply to Rohlf.. Syst Biol.

[24] Zelditch ML, Fink WL (1995). Allometry and developmental integration of body growth in a piranha, *Pygocentrus nattereri* (Teleostei: Ostariophysi).. J Morphol.

[25] Reis RE, Zelditch ML, Fink WL (1998). Ontogenetic allometry of body shape in the Neotropical catfish Callichthys (Teleostei: Siluriformes).. Copeia.

[26] Wu RL, Lin M (2006). Functional mapping - How to map and study the genetic architecture of dynamic complex traits.. Nat Rev Genet.

[27] The International HapMap Consortium (2003). The International HapMap Project.. Nature.

[28] Lander ES, Botstein D (1989). Mapping Mendelian factors underlying quantitative traits using RFLP linkage maps.. Genetics.

[29] Zeng ZB (1993). Theoretical basis for separation of multiple linked gene effects in mapping quantitative trait loci.. Proc Natl Acad Sci USA.

[30] Zeng ZB (1994). Precision mapping of quantitative trait loci.. Genetics.

[31] Lynch M, Walsh B (1998). Genetics and Analysis of Quantitative Traits.

[32] Kao C-H, Zeng Z-B, Teasdale RD (1999). Multiple interval mapping for quantitative trait loci.. Genetics.

[33] Broman KW, Speed TP (2002). A model selection approach for the identification of quantitative trait loci in experimental crosses (with discussion).. J Roy Stat Soc B.

[34] Yi NJ, Yandell B, Churchill G, Allison D, Eisen EJ, Pomp D (2005). Bayesian model selection for genome-wide epistatic analysis.. Genetics.

[35] Yi NJ, Shriner D, Banerjee S, Mehta T, Pomp D, Yandell BS (2007). An efficient Bayesian model selection approach for interacting quantitative trait loci models with many effects.. Genetics.

[36] Wu RL, Lin M, Zhao W, Hou W, Zhang B, Zhuge Q, Huang MR, Xu LA (2006). Statistical models for studying the genetic architecture of dynamic complex traits.. J Nanjing Forestry Univ.

[37] Jin CF, Fine JP, Yandell BS (2007). A unified semiparametric framework for quantitative trait loci analyses, with application to spike phenotypes.. J Am Stat Assoc.

[38] Jiang C, Zeng Z-B (1995). Multiple trait analysis of genetic mapping for quantitative trait loci.. Genetics.

[39] Knott SA, Haley CS (2000). Multitrait least squares for quantitative trait loci detection.. Genetics.

[40] Vieira C, Pasyukova EG, Zeng ZB, Hackett JB, Lyman RF, Mackay TFC (2000). Genotype environment interaction for quantitative trait loci affecting life span in *Drosophila melanogaster*.. Genetics.

[41] Korol AB, Ronin YI, Itskovich AM, Peng J, Nevo E (2001). Enhanced efficiency of quantitative trait loci mapping analysis based on multivariate complexes of quantitative traits.. Genetics.

[42] Shaw RG (1987). Maximum-likelihood approaches applied to quantitative genetics of natural populations.. Evolution.

[43] Wu RL, Ma CX, Littell RC, Casella G (2002). A statistical model for the genetic origin of allometric scaling laws in biology.. J Theor Biol.

[44] Ma CX, Casella G, Littell RC, Khuri AI, Wu RL (2003). Exponential mapping of quantitative traits governing allometric relationships in organisms.. J Math Biol.

[45] Wu RL, Hou W (2006). A hyperspace model to decipher the genetic architecture of developmental processes: Allometry meets ontogeny.. Genetics.

[46] Long F, Chen YQ, Cheverud JM, Wu RL (2006). Genetic mapping of allometric scaling laws.. Genet Res.

[47] Ma CX, Casella G, Wu RL (2002). Functional mapping of quantitative trait loci undelying the character process: A theoretical framework.. Genetics.

[48] Jiang C, Zeng Z-B (1997). Mapping quantitative trait loci with dominant and missing markers in various crosses from two inbred lines.. Genetica.

[49] Diggle PJ, Heagerty P, Liang KY, Zeger SL (2002). Analysis of Longitudinal Data.

[50] Núñez-Antón V, Zimmerman DL (2000). Modeling nonstationary longitudinal data.. Biometrics.

[51] Zimmerman DL, Núñez-Antón V (2001). Parametric modeling of growth curve data: An overview (with discussion).. Test.

[52] Louis TA (1982). Finding the observed information matrix when using the EM algorithm.. J Roy Stat Soc B.

[53] Wu RL, Ma CX, Lin M, Casella G (2004). A general framework for analyzing the genetic architecture of developmental characteristics.. Genetics.

[54] Churchill GA, Doerge RW (1994). Empirical threshold values for quantitative trait mapping.. Genetics.

[55] Niklas KJ, Enquist BJ (2001). Invariant scaling relationships for interspecific plant biomass production rates and body size.. Proc Natl Acad Sci USA.

[56] Zhang WK, Wang YJ, Luo GZ, Zhang JS, He CY, Wu XL, Gai JY, Chen SY (2004). QTL mapping of ten agronomic traits on the soybean (*Glycine*).. Theor Appl Genet.

[57] Wu RL, Ma C-X, Casella G (2007). Statistical Genetics of Quantitative Traits: Linkage, Maps, and QTL.

[58] Klingenberg CP, Zimmermann M (1992). Static, ontogenetic, and evolutionary allometry: A multivariate comparison in nine species of water striders.. Am Nat.

[59] Breuker C, Debat V, Klingenberg CP (2006). Functional evo-devo.. Trends Ecol Syst.

[60] Yap JS, Wang C, Wu R (2007). A computational approach for functional mapping of quantitative trait loci that regulate thermal performance curves.. PLoS ONE.

[61] von Bertalanffy L (1957). Quantitative laws for metabolism and growth.. Quart Rev Biol.

[62] West GB, Brown JH, Enquist BJ (2001). A general model for ontogenetic growth.. Nature.

[63] Hou W, Garvan CW, Zhao W, Behnke M, Eyler FD, Wu RL (2005). A generalized model for detecting genetic determinants underlying longitudinal traits with unequally spaced measurements and time-dependent correlated errors.. Biostatistics.

[64] Zhao W, Zhu J, Gallo-Meagher M, Wu RL (2004). A unified statistical model for functional mapping of genotype×environment interactions for ontogenetic development.. Genetics.

