# Supplementary material for: A Conceptual Framework for Mapping Quantitative Trait Loci Regulating Ontogenetic Allometry
Source: PLoS One. 2007 Nov 28;2(11):e1245. doi: 10.1371/journal.pone.0001245 (PMC2080758; doi:10.1371/journal.pone.0001245)
Supplement: Appendix S1 — (0.09 MB DOC) [file pone.0001245.s001.doc]

**Appendix**

The EM algorithm is a powerful numerical technique for finding maximum likelihood estimates (MLEs) in standard incomplete data problems. Below, we provide the EM algorithm for estimating the QTL genotype-specific curve parameters $\Omega_{q}=\left(\alpha_{j},\beta_{j}\right)$, and $\Omega_{v}=\left(\rho,\sigma^{2}\right)$ that model the covariance matrix structure with the SAD(1) model. As shown in Equation 3, the observed log-likelihood function is

$$\begin{matrix} \log L(\text{y},\text{M})=\mathop{\Sigma}\limits_{i=1}^{n} \log\left\{\omega_{1|i}(2\pi)^{-\frac{T}{2}}|\Sigma|^{-\frac{1}{2}}\exp\left[-\frac{1}{2}(\text{y}_{i}-\text{u}_{1|i})^{'}\Sigma^{-1}(\text{y}_{i}-\text{u}_{1|i})\right]\right. \\ \text{ +}\left.\omega_{2|i}(2\pi)^{-\frac{T}{2}}|\Sigma|^{-\frac{1}{2}}\exp\left[-\frac{1}{2}(\text{y}_{i}-\text{u}_{2|i})^{'}\Sigma^{-1}(\text{y}_{i}-\text{u}_{2|i})\right]\right\} \end{matrix}$$

$\text{u}_{1|i}=\left(\alpha_{1}+\beta_{1}z_{i}(t_{1}),\alpha_{1}+\beta_{1}z_{i}(t_{2}),\ldots,\alpha_{1}+\beta_{1}z_{i}(t_{T})\right)^{'},$where

$$\text{u}_{2|i}=\left(\alpha_{2}+\beta_{2}z_{i}(t_{1}),\alpha_{2}+\beta_{2}z_{i}(t_{2}),\ldots,\alpha_{2}+\beta_{2}z_{i}(t_{T})\right)^{'}.$$

Now, suppose the missing QTL genotype $\text{Q}_{i}$ for each individual is observed. Then, the complete log-likelihood function is written as follows:

$$\begin{matrix} \log L_{c}\left(\text{y},\text{Q}\right)=\sum_{i=1}^{n} \log\left\{\left(2\pi\right)\right.^{-\frac{T}{2}}\left|\Sigma\right|^{-\frac{1}{2}}\exp\left[-\frac{1}{2}\right.\left(\text{y}_{i}-\text{u}_{1|i}I(\text{Q}_{i}=1)-\text{u}_{2|i}I(\text{Q}_{i}=2)\right)^{'} \\ \text{ }\left.\left.\text{ }\Sigma^{\text{-1}}\left(\text{y}_{i}-\text{u}_{1|i}I(\text{Q}_{i}=1)-\text{u}_{2|i}I(\text{Q}_{i}=2)\right)\right]\right\} \\ \text{ }=-\frac{nT}{2}\log\left(2\pi\right)-\frac{n}{2}\log\left|\Sigma\right|-\frac{1}{2}\sum_{i=1}^{n} \left[\left(\text{y}_{i}-\text{u}_{1|i}\right)^{'}\Sigma^{-1}\left(\text{y}_{i}-\text{u}_{1|i}\right)\right]I(\text{Q}_{i}=1) \\ \text{ }-\frac{1}{2}\sum_{i=1}^{n} \left[\left(\text{y}_{i}-\text{u}_{2|i}\right)^{'}\Sigma^{-1}\left(\text{y}_{i}-\text{u}_{2|i}\right)\right]I(\text{Q}_{i}=2) \end{matrix}$$

In the E-step of the EM algorithm, we calculate the conditional expectation of the complete log-likelihood given the observed data and the current estimation of parameters. We define the E-step by expressing the posterior probabilities of individual $i$ to be QTL genotype *QQ* (1) or *qq* (2) as

$$\begin{matrix} \Omega_{1|i}=\frac{\omega_{1|i}\left(2\pi\right)^{-\frac{T}{2}}\left|\Sigma\right|^{-\frac{1}{2}}\exp\left[-\frac{1}{2}\left(\text{y}_{i}-\text{u}_{1|i}\right)^{'}\Sigma^{-1}\left(\text{y}_{i}-\text{u}_{1|i}\right)\right]}{\omega_{1|i}\left(2\pi\right)^{-\frac{T}{2}}\left|\Sigma\right|^{-\frac{1}{2}}\exp\left[-\frac{1}{2}\left(\text{y}_{i}-\text{u}_{1|i}\right)^{'}\Sigma^{-1}\left(\text{y}_{i}-\text{u}_{1|i}\right)\right]+\omega_{2|i}\left(2\pi\right)^{-\frac{T}{2}}\left|\Sigma\right|^{-\frac{1}{2}}\exp\left[-\frac{1}{2}\left(\text{y}_{i}-\text{u}_{2|i}\right)^{'}\Sigma^{-1}\left(\text{y}_{i}-\text{u}_{2|i}\right)\right]} \\ \Omega_{2|i}=\frac{\omega_{2|i}\left(2\pi\right)^{-\frac{T}{2}}\left|\Sigma\right|^{-\frac{1}{2}}\exp\left[-\frac{1}{2}\left(\text{y}_{i}-\text{u}_{2|i}\right)^{'}\Sigma^{-1}\left(\text{y}_{i}-\text{u}_{2|i}\right)\right]}{\omega_{1|i}\left(2\pi\right)^{-\frac{T}{2}}\left|\Sigma\right|^{-\frac{1}{2}}\exp\left[-\frac{1}{2}\left(\text{y}_{i}-\text{u}_{1|i}\right)^{'}\Sigma^{-1}\left(\text{y}_{i}-\text{u}_{1|i}\right)\right]+\omega_{2|i}\left(2\pi\right)^{-\frac{T}{2}}\left|\Sigma\right|^{-\frac{1}{2}}\exp\left[-\frac{1}{2}\left(\text{y}_{i}-\text{u}_{2|i}\right)^{'}\Sigma^{-1}\left(\text{y}_{i}-\text{u}_{2|i}\right)\right]} \end{matrix}$$

The M step is derived by solving the log-likelihood equations of the expected complete log-likelihood function given the observed data and current estimations. These equations lead to

$$\hat{\alpha}_{1}=\frac{\left(\sum_{i=1}^{n} \Omega_{1|i}\text{y}_{i}^{'}\Sigma^{-1}1\right)\left(\sum_{i=1}^{n} \Omega_{1|i}\text{z}_{i}^{'}\Sigma^{-1}\text{z}_{i}\right)-\left(\sum_{i=1}^{n} \Omega_{1|i}\text{y}_{i}^{'}\Sigma^{-1}\text{z}_{i}\right)\left(\sum_{i=1}^{n} \Omega_{1|i}\text{z}_{i}^{'}\Sigma^{-1}1\right)}{\left(\sum_{i=1}^{n} \Omega_{1|i}1^{'}\Sigma^{-1}1\right)\left(\sum_{i=1}^{n} \Omega_{1|i}\text{z}_{i}^{'}\Sigma^{-1}\text{z}_{i}\right)-\left(\sum_{i=1}^{n} \Omega_{1|i}1^{'}\Sigma^{-1}\text{z}_{i}\right)^{2}}$$

$$\begin{matrix} \hat{\alpha}_{2}=\frac{\left(\sum_{i=1}^{n} \Omega_{2|i}\text{y}_{i}^{'}\Sigma^{-1}1\right)\left(\sum_{i=1}^{n} \Omega_{2|i}\text{z}_{i}^{'}\Sigma^{-1}\text{z}_{i}\right)-\left(\sum_{i=1}^{n} \Omega_{2|i}\text{y}_{i}^{'}\Sigma^{-1}\text{z}_{i}\right)\left(\sum_{i=1}^{n} \Omega_{2|i}\text{z}_{i}^{'}\Sigma^{-1}1\right)}{\left(\sum_{i=1}^{n} \Omega_{2|i}1^{'}\Sigma^{-1}1\right)\left(\sum_{i=1}^{n} \Omega_{2|i}\text{z}_{i}^{'}\Sigma^{-1}\text{z}_{i}\right)-\left(\sum_{i=1}^{n} \Omega_{2|i}1^{'}\Sigma^{-1}\text{z}_{i}\right)^{2}} \\ \hat{\beta}_{1}=\frac{\left(\sum_{i=1}^{n} \Omega_{1|i}\text{y}_{i}^{'}\Sigma^{-1}1\right)\left(\sum_{i=1}^{n} \Omega_{1|i}1^{'}\Sigma^{-1}\text{z}_{i}\right)-\left(\sum_{i=1}^{n} \Omega_{1|i}\text{y}_{i}^{'}\Sigma^{-1}\text{z}_{i}\right)\left(\sum_{i=1}^{n} \Omega_{1|i}1^{'}\Sigma^{-1}1\right)}{\left(\sum_{i=1}^{n} \Omega_{1|i}1^{'}\Sigma^{-1}\text{z}_{i}\right)^{2}-\left(\sum_{i=1}^{n} \Omega_{1|i}1^{'}\Sigma^{-1}1\right)\left(\sum_{i=1}^{n} \Omega_{1|i}\text{z}_{i}^{'}\Sigma^{-1}\text{z}_{i}\right)} \\ \hat{\beta}_{2}=\frac{\left(\sum_{i=1}^{n} \Omega_{2|i}\text{y}_{i}^{'}\Sigma^{-1}1\right)\left(\sum_{i=1}^{n} \Omega_{2|i}1^{'}\Sigma^{-1}\text{z}_{i}\right)-\left(\sum_{i=1}^{n} \Omega_{2|i}\text{y}_{i}^{'}\Sigma^{-1}\text{z}_{i}\right)\left(\sum_{i=1}^{n} \Omega_{2|i}1^{'}\Sigma^{-1}1\right)}{\left(\sum_{i=1}^{n} \Omega_{1|i}1^{'}\Sigma^{-1}\text{z}_{i}\right)^{2}-\left(\sum_{i=1}^{n} \Omega_{1|i}1^{'}\Sigma^{-1}1\right)\left(\sum_{i=1}^{n} \Omega_{1|i}\text{z}_{i}^{'}\Sigma^{-1}\text{z}_{i}\right)} \end{matrix}$$

$$\begin{matrix} \hat{\rho}=\frac{\sum_{i=1}^{n} \left[\Omega_{1|i}\left(\text{y}_{i}-\text{u}_{1|i}\right)^{'}\text{B}_{1}\left(\text{y}_{i}-\text{u}_{1|i}\right)+\Omega_{2|i}\left(\text{y}_{i}-\text{u}_{2|i}\right)^{'}\text{B}_{1}\left(\text{y}_{i}-\text{u}_{2|i}\right)\right]}{\sum_{i=1}^{n} \left[\Omega_{1|i}\left(\text{y}_{i}-\text{u}_{1|i}\right)^{'}\text{B}_{2}\left(\text{y}_{i}-\text{u}_{1|i}\right)+\Omega_{2|i}\left(\text{y}_{i}-\text{u}_{2|i}\right)^{'}\text{B}_{2}\left(\text{y}_{i}-\text{u}_{2|i}\right)\right]} \\ \hat{\sigma}^{2}=\frac{1}{nT}\sum_{i=1}^{n} \left[\Omega_{1|i}\left(\text{y}_{i}-\text{u}_{1|i}\right)^{'}\text{A}\left(\text{y}_{i}-\text{u}_{1|i}\right)+\Omega_{2|i}\left(\text{y}_{i}-\text{u}_{2|i}\right)^{'}\text{A}\left(\text{y}_{i}-\text{u}_{2|i}\right)\right] \end{matrix}$$

where $1$is a T × 1 vector of 1’s, and

$$\begin{matrix} \text{A}=\sigma^{2}\Sigma^{-1} \\ \text{B}_{1}=\left[\begin{matrix} 0 & 1 & 0 & \cdots& 0 & 0 \\ 1 & 0 & 1 & \cdots& 0 & 0 \\ 0 & 1 & 0 & \cdots& 0 & 0 \\ \vdots& \vdots& \vdots& \cdots& \vdots& \vdots\\ 0 & 0 & 0 & \cdots& 1 & 0 \end{matrix}\right] \\ \text{B}_{2}=\text{diag}\left\{\text{2,}\ldots\text{,2,0}\right\} \end{matrix}$$

The E and M steps are iteratively repeated until the estimates of parameters are stable. These stable estimates are regarded as the MLEs of parameters. The estimates of the sampling errors for the MLEs are obtained using Louis’ [52] approach.
